# Parental Praise Correlates with Posterior Insular Cortex Gray Matter Volume in Children and Adolescents

**DOI:** 10.1371/journal.pone.0154220

**Published:** 2016-04-21

**Authors:** Izumi Matsudaira, Susumu Yokota, Teruo Hashimoto, Hikaru Takeuchi, Kohei Asano, Michiko Asano, Yuko Sassa, Yasuyuki Taki, Ryuta Kawashima

**Affiliations:** 1 Department of Nuclear Medicine & Radiology, Institute of Development, Aging and Cancer, Tohoku University, Sendai, Japan; 2 Division of Developmental Cognitive Neuroscience, Institute of Development, Aging and Cancer, Tohoku University, Sendai, Japan; 3 Division of Medical Neuroimaging Analysis, Department of Community Medical Supports, Tohoku Medical Megabank Organization, Tohoku University, Sendai, Japan; 4 Department of Functional Brain Imaging, Institute of Development, Aging, and Cancer, Tohoku University, Sendai, Japan; 5 Smart Aging International Research Center, Institute of Development, Aging, and Cancer, Tohoku University, Sendai, Japan; Banner Alzheimer's Institute, UNITED STATES

## Abstract

A positive parenting style affects psychological and cognitive development in children. Neuroimaging studies revealed that a positive parenting style influenced brain structure in children. Parental praise is a concrete behavior observed in positive parenting. Although previous psychological studies revealed a positive effect of parental praise on children, little is known about the relationship between parental praise and brain structure in children. Thus, the purpose of the present study was to determine whether there was a correlation between the parental attitude towards praising their child and gray matter volume in the children (116 boys and 109 girls; mean age, 10.6 years old). We examined the correlation between regional gray matter volume and parental praise using voxel-based morphometry (VBM) following magnetic resonance imaging (MRI). In addition, to confirm the positive effects of parental praise, we analyzed the correlation between the frequency of parental praise and personality traits in children. We showed that the parental attitude towards praising their child was significantly and positively correlated with the gray matter volume of the left posterior insular cortex in children. Moreover, we found a significant positive correlation between parental attitude towards praising their child and the personality traits of conscientiousness and openness to experience in the children. Prior studies said that gray matter volume in the posterior insula was correlated with empathy, and the functional connectivity between this area and the amygdala was associated with emotional regulation. Furthermore, the posterior insula relates to auditory function, and therefore, was likely involved in the processing of parental praise. Considering the possibility of experience-dependent plasticity, frequent parental praise would lead to increased posterior insular gray matter volume in children. Our study is the first to elucidate the relationship between a specific positive parenting behavior and brain structure in children.

## Introduction

Parenting style affects the development of children. In fact, positive parenting leads to positive psychological outcomes in children. For example, socialization [1] and psychological well-being [2] in children is related to positive parenting. In addition, personality traits in children are also influenced by parenting. For example, children who perceived that their parents were accepting were more extraverted, open to experience, agreeable, and conscientious [3]. Further, adults who experienced less parental care in their childhood were more neurotic and less conscientious [4]. Consequently, it is thought that positive parenting influences psychological development in children.

The relationship between parenting and brain structure in children has been studied. Whittle et al. (2014) showed that the volume of the amygdala, a brain region associated with emotional hypersensitivity, was negatively correlated with positive maternal behavior [5]. Luby et al. (2012) found that early maternal support predicted larger hippocampal volume in school-age children, particularly in non-depressed, healthy children [6]. Moreover, Sheikh et al. (2014) revealed that positive parental caregiving affected the development of white matter fractional anisotropy in brain regions related to the stress response, particularly in the region associated with cortisol reactivity [7]. These studies show that positive parenting can affect brain structure in children. Similarly, negative parenting, which is equivalent to maltreatment, affects brain structure in children. For instance, gray matter has been found to develop irregularly in the superior temporal gyrus of young adults who experienced parental verbal abuse [8].

Parental praise represents a positive expression of social feedback and verbal reward for children [9]. Parents utilize this behavior to improve their child’s self-esteem, self-efficacy, and motivation [10,11], and this behavior can also contribute to the development of a warm environment and promotion of attachment [9]. Previous studies investigating the effectiveness of parental praise have indicated that it is associated with several elements of positive parenting including loving and responsive care [12], supportiveness [13], warmth, and positive affect [9]. In fact, a recent study determined that these elements may also be used, at least in part, to define positive parenting [14]. Therefore, parental praise may be regarded as one aspect of positive parenting behavior. n. Furthermore, several studies have revealed that parental praise influences a number of variables associated with children’s outcomes, including self-esteem [13], motivation [15], emotional and physical well-being [16], and social competence [17]. It has also been shown that, as a verbal stimulus, parental praise can influence language development in children. For example, children with language delays typically receive significantly less parental praise than do children with adequate language skills in economically disadvantaged families [18]. Therefore, parental praise is associated with psychological and cognitive development in children.

However, to the best of our knowledge, few neuroimaging studies have attempted to elucidate the effects of parental praise on brain structure in children. As mentioned above, prior neuroimaging studies have revealed that positive parenting influences the brain structure of children [5–7], but the specific behaviors performed by parents during positive parenting that are effective for promoting brain structure in children remain unclear. Therefore, the purpose of this study was to investigate the positive effects of parental praise on brain structure in children.

In this study, we conducted a voxel-based morphometry (VBM) analysis to clarify the correlation between parental attitude towards praising their child and regional gray matter volume in children and adolescents using magnetic resonance imaging (MRI). Based on previous studies showing a positive effect of parenting on amygdala and hippocampal structures [5,6], it was hypothesized that the gray matter volumes of the amygdala, hippocampus, or brain regions associated with these areas would be significantly correlated with parental attitude towards praising their child. Additionally, we examined the correlation between parental attitude towards praising their child and personality traits in the children to confirm the positive effects of parental praise on psychological outcomes that have been reported previously[13,15–17].

## Material and Methods

### Subjects

The study participants were healthy Japanese children. The details regarding their initial recruitment was described previously [19,20]. In brief, we obtained brain magnetic resonance (MR) images and various behavioral data from 290 children (145 boys and 145 girls; age range, 5.6–18.4 years) at Time Point 1. No participant had a history of malignant tumors or head injuries involving a loss of consciousness. Subject recruitment materials specified that only right-handed children were eligible for the study. Additionally, the Edinburgh Handedness Inventory, a self-report questionnaire, was used to confirm right-handedness [21]. The study was conducted according to the Declaration of Helsinki (1991), and written informed consent was obtained from each participant and his or her parent prior to MR scanning and after a full explanation of the purpose of and procedures used in the study. A special version of informed consent was obtained from the children. Our study was approved by the Institutional Review Board of Tohoku University. The Time Point 2 follow-up study, which included 235 children, was conducted approximately 3 years after the Time Point 1 study.

For all subject data used in the analysis, parental praise data and MR images were collected at Time Point 1; personality trait data were acquired at Time Point 2. The number of subjects included was 225 (116 boys and 109 girls; age range at Time Point 1: 5.6–18.4 years) (Table 1).

**Table 1:** Characteristics of the study participants.

	Mean	SD	Minimum	Maximum
Age	10.64	3.04	5	18
Gender (male: female)	116:109			
Full-scale IQ	103.46	11.94	71	133
Parents' educational background ^a^	14.22	1.65	9.0	18.5
Parental praise score ^b^	4.08	0.88	1	5
Extraversion ^c^	-0.014	1.004	-1.99	1.23
Agreeableness ^c^	-0.023	0.990	-2.59	1.80
Conscientiousness ^c^	0.001	1.004	-1.61	2.06
Neuroticism ^c^	-0.002	0.998	-2.31	2.03
Openness ^c^	0.002	1.015	-1.63	2.38

^a^: Parents’ educational background was categorized as follows: 1, elementary school graduate or below; 2, junior high school graduate; 3, normal high school graduate; 4, graduate of a short-term school completed after high school (such as a junior college); 5, university graduate; 6, master’s degree; and 7, doctorate. The average of the answers provided by the parents was used in the analyses.

^b^: The parental praise score is the rating the parent chose for the question; “You try to praise your child for his or her good point.”

^c^: The raw personality trait scores of the participants were converted to standard scores because the number of question items differed between the two types of tests.

Although the present study collected and analyzed data from the Child Behavior Checklist, none of the subjects were excluded based on their score on this test in this project [19,20,22,23]. This is partly because this criterion would have resulted in exclusion of many subjects who do not require treatment at a hospital for the particular issues they suffer from. Furthermore, a high degree of continuity between normality and abnormality has been consistently shown for these measures [24]. Therefore, there were no practical reasons to exclude any subjects based on this score. Nevertheless, to determine whether this procedure affected the results and conclusions of the present study, the data were analyzed without including subjects who received a T-score > 70 on any subscale from the Child Behavior Checklist [25]. The use of this criterion resulted in the exclusion of additional 18 subjects, and the subsequent analysis demonstrated that the primary significant results of the present study did not change significantly.

### Assessment of behavioral data

#### Parental praise

Parents answered a question regarding their attitude towards praising their child, using a Likert scale ranging from 1–5 (strongly disagree to strongly agree) that corresponded to the extent with which they disagreed or agreed with the item. We treated the number that parents chose as the parental praise score. The question presented to the parents was as follows: “You try to praise your child for his or her good point. This question was an item of the Family Diagnostic Test (FDT) [26]. We decided to use this question only because we were particularly interested in the influence of parental praise on their children’s development, as various studies showed that parental praise affected the trajectory of child development in a positive manner. We sent this question via post at Time Point 1; therefore, parents answered this question before MR images of the children were acquired.

#### Personality traits

Children were administered the personality trait questionnaire at Time Point 2. Depending on participant age, the Little Big Five Personality Inventory[27] or Big Five Personality Inventory[28] was used to assess the personality traits of children. We used the former for elementary school children. The latter was used for children of junior high school age and older, as it was established for adults. The consistency of the constructs in these tests was confirmed by Murakami and Hatayama (2010). The test for adults consisted of 70 items, and the test for children was comprised of 47 items. Both tests were based on the Big Five, which is rooted in the lexical approach[29]. These are self-report questionnaires in which participants had to choose “yes” or “no” regarding whether their personality traits corresponded to the statements. This inventory provided scores for five dimensions of personality: extraversion, agreeableness, conscientiousness, neuroticism, and openness to experience. These questionnaires were also sent by post; therefore, the subjects completed it prior to MR image acquisition at Time Point 2.

#### IQ test

IQ was measured using the Japanese version of the age-appropriate Wechsler test, which was administered on the same day as the MRI scans at time-points 1 and 2. The Wechsler Intelligence Scale for Children, Third edition (WISC–III) was used for subjects younger than 16 years, and the Wechsler Adult Intelligence Scale, Third edition (WAIS–III) was used for subjects older than 16 years [30–32].

#### Parents’ educational background

In the present study, the parents of the subjects were asked describe their educational background using the following categories: 1, elementary school graduate or below; 2, junior high school graduate; 3, normal high school graduate; 4, graduate of a short-term school completed after high school (such as a junior college); 5, university graduate; 6, master’s degree; and 7, doctorate. The average of the answers provided by the parents was used in the analyses.

#### Image acquisition

All images were collected using 3T Philips Intera Achieva scanner at Time Point 1. Using a magnetization-prepared rapid gradient-echo (MPRAGE) sequence, three-dimensional (3D), high-resolution, T1-weighted images (T1WIs) were collected. The parameters were as follows: 240 × 240 matrix, TR = 6.5 ms, TE = 3 ms, TI = 711 ms, FOV = 24 cm, 162 slices, 1.0-mm slice thickness, and scan duration of 8 minutes and 3 seconds.

#### Pre-processing of images

Pre-processing of the structural images was performed using Statistical Parametric Mapping software (SPM8; Wellcome Department of Cognitive Neurology, London, UK) implemented in MATLAB (Mathworks Inc., Natick, MA, USA). Using the new segmentation algorithm implemented in SPM8, T1WIs from each subject were segmented into six tissues. During this process, the gray matter tissue probability map (TPM) used in this procedure was manipulated from maps implemented in the software so that the signal intensities of the voxels (gray matter tissue probability of the default tissue gray matter TPM + white matter tissue probability of the default TPM) <0.25 became 0. When this manipulated gray matter TPM was used, the dura mater was less likely to be classified as gray matter (compared with when the default gray matter TPM was used) without other substantial segmentation problems. In this new segmentation process, the default parameters were used, with the exception of affine regularization, which was performed using the International Consortium for Brain Mapping (ICBM) template for East Asian brains.

We then conducted diffeomorphic anatomical registration through exponentiated lie algebra (DARTEL) registration process implemented in SPM8. During this process, we used DARTEL-imported images of the six gray matter TPMs created using the abovementioned new segmentation process. First, the template for the DARTEL procedure was created using the T1WI data from the Time Point 1 scan from all subjects. Next, using this existing template, DARTEL procedures were performed using the scans acquired at Time Point 2 for all participants included in this study and the default parameter settings.

The resulting images were then spatially normalized to the Montreal Neurological Institute (MNI) space to obtain images of 1.5 × 1.5 × 1.5 mm^3^ voxels. Subsequently, all images were smoothed by convolving them with an 8-mm full width at half-maximum (FWHM) isotropic Gaussian kernel.

#### Statistical analysis

Behavioral data were analyzed using IBM SPSS Statistics 21 software. Partial correlation analyses were conducted to investigate the relationships between the parental praise score and the children’s personality trait variables, using the age, gender, and full-scale IQ of the children and the parents’ educational background as covariates. Results with a threshold of *p< 0*.*05* were considered to be statistically significant. Prior to the analysis, we converted raw personality trait scores to standard scores because the number of question items differed between the two types of tests.

We performed statistical brain structural analysis using SPM8. We conducted multiple regression analysis in SPM8 to determine the correlation between the parental praise score and the regional gray matter volume in children. In this analysis, each regional gray matter volume was set as a dependent variable and the parental praise score was used as a covariate of interest. The age, gender, and full-scale IQ, and intracranial volume of children and the parents’ educational background were added as covariates of no interest. In the whole brain imaging analysis, the level of statistical significance was set at *p < 0*.*05*, and corrected at the non-isotropic adjusted cluster level using family wise error (FWE) with an underlying voxel-level *p < 0*.*001*. Non-isotropic adjusted cluster-size tests can and should be applied when cluster-size tests are applied to data known to be non-stationary (i.e., not uniformly smooth), such as VBM data [33].

## Results

### Behavioral results

The parental praise score was significantly and positively correlated with conscientiousness (r = 0.200, *p* = 0.003) and openness (r = 0.191, *p* = 0.004) in children (Table 2). No other personality trait was significantly correlated with the parental praise score.

**Table 2:** Partial correlations (Pearson’s *r*) between parental praise and personality traits in children.

	Extraversion	Agreeableness	Conscientiousness	Neuroticism	Openness
Parental praise score	0.05	-0.024	0.200**	0.014	0.191**

**: *P* < 0.01

Adjusted for the age, gender, and full-scale IQ of the children and the parents’ educational background.

### Imaging results

**Fig 1:**
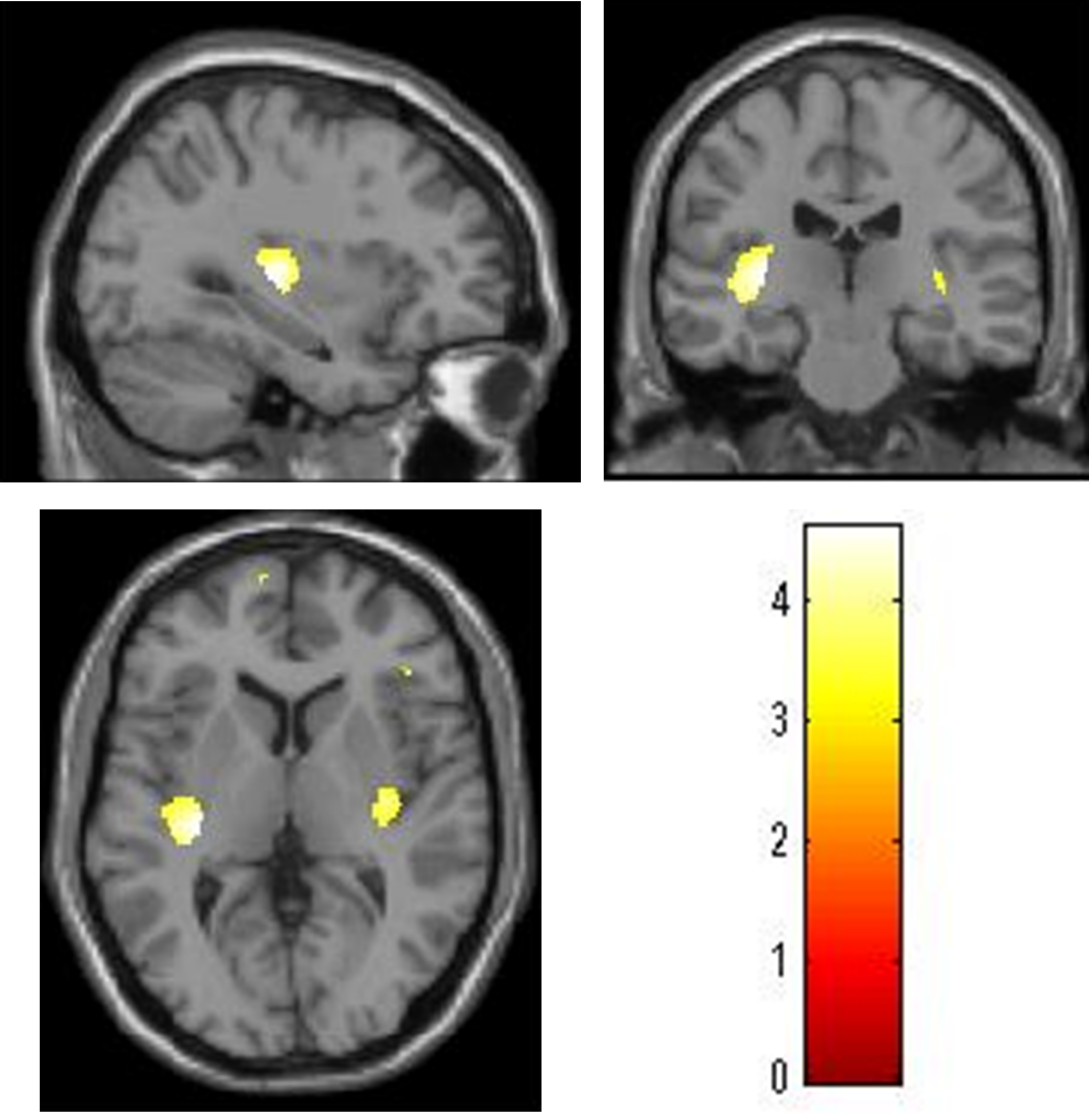
Regions in which gray matter volume was positively correlated with the parental praise score. We adjusted for age, sex, full-scale IQ, and total intracranial volume of the children, and the parents’ educational background. The left side of the image corresponds to the left side of the brain. The color scale indicates the *t*-scores. We adjusted the figure to account for the reduced statistical threshold to *P* < 0.001 uncorrected for visibility.

The gray matter volume of the left posterior insular cortex (x, y, z, = -32, -22, 4; -41, -15, -2; *k* = 972, *P* = 0.025, FWE corrected at the cluster level) was significantly and positively correlated with the parental praise score, after adjusting for age, gender, full-scale IQ, and total intracranial volume of the children, and the parents’ educational background in the multiple regression analysis (Table 3, Fig 1). The positive correlation between the parental praise score and the gray matter volume of a similar area in the right hemisphere was also revealed under the liberal statistical threshold (*P <* 0.001, uncorrected).

**Table 3:** Brain regions and MNI coordinates showing significant positive correlations with the parental praise score.

Brain area	Peak voxel				Cluster size	*P* value
	MNI coordinates			*T* score		
	x	y	z			
L-posterior insula	-32	-22	4	4.63	972	0.025
L-posterior insula	-41	-15	-2	4.51	972	0.025

The level of statistical significance was set at *p < 0*.*05*, and corrected at the non-isotropic adjusted cluster level using family wise error (FWE) with an underlying voxel-level *p < 0*.*001*. We adjusted for age, gender, intracranial volume, and full-scale IQ of the children and the parents’ educational background.

## Discussion

The present findings demonstrated that parental praise was associated with the brain structure and personality traits of children. Neuroimaging data illustrated that the parental praise score significantly and positively correlated with gray matter volume of the left posterior insular cortex. In addition, the behavioral data demonstrated a significant and positive correlation between the parental praise score and personality traits (i.e., conscientiousness and openness to experience).

In general, the posterior insula is associated with somatic sensation [34]. Furthermore, the region is linked to interoceptive function via interactions between the brain and body [35]. The capacity for interoception is correlated with the ability to understand the emotions of others [36], and indeed, the posterior insular cortex has been shown to be involved in emotional functioning. For example, previous studies have shown that greater gray matter volume in the posterior insula is associated with guilty feelings, which are related to empathy [37], and that there is a reduction in gray matter volume in patients with cognitive alexithymia [38]. Because greater gray matter volume in the posterior insula is associated with the recognition of self and of other’s emotional status, it is reasonable to expect that praising a child frequently would facilitate the development of empathy.

Functional connectivity between the posterior insula and amygdala may explain the correlation between the parental praise score and posterior insular cortex gray matter volume. The posterior insula is functionally connected to the amygdala, and this connectivity relates to emotional regulation [39]. Denny et al. (2015) reported that the resting-state functional connectivity between the posterior insula and amygdala decreased with the severity of emotional dysregulation and depression in young people. Positive parenting has been shown to affect the structure of the amygdala, leading to the speculation that positive parenting may mediate emotional regulation in adolescents[5]. These findings suggest that parental praise, a positive parenting behavior, affects the structure of the posterior insular cortex, which is functionally connected to the amygdala.

The correlation between the parental attitude towards praising their child and gray matter volume in the posterior insula, but not in the amygdala, may be attributable to the auditory function of the posterior insula. Cytoarchitectural studies have shown that the posterior insula is connected to the auditory cortex [40], and that it interacts with various brain regions, including the primary auditory cortex via fiber pathways [41]. Functional neuroimaging studies have also revealed that the posterior insula supports auditory function. For instance, the posterior insula shows higher activation in response to a biological human voice than to an artificial voice [42]. Furthermore, in an auditory language task in which participants were required to identify common words from a phrase describing the words (e.g., “jewelry we wear around our neck”), posterior insula activation was significantly higher in typically developing subjects than in subjects with autism spectrum disorder [43]. The authors interpreted this finding as evidence for posterior insula involvement in the processing of emotional aspects of language. Parental praise is primarily a verbal auditory stimulus that includes parental emotions such as affection and warmth [44,45]. Thus, it is possible that parental praise affects the posterior insula preferentially to the amygdala.

The question item used in the present study to indicate parental attitude towards praising their child was “You try to praise your child for his or her good point”, and it was assumed that the parental attitude towards praising their child would reflect the frequency of parental praise. We surmise that frequent parental praise, as verbal emotional stimuli, leads to repeated activation of the posterior insular cortex. Experience-dependent plasticity has not been demonstrated in the posterior insula; however, anterior and mid-insula plasticity has been reported. Gray matter volume in the anterior insula is positively correlated with the frequency of expressive suppression, an aspect of emotion regulation [46]. Moreover, a positive correlation has been reported between gray matter volume in the mid-insula and years of yoga experience [47]. Although the subdivisions of the insular cortex have different functions [48] and cytoarchitectonic structures [40,49], functional connections exist among the regions [50,51]. Thus, it is possible that experience-dependent plasticity occurs in the posterior insula. From this perspective, we propose that frequent parental praise leads to repeated activation of the posterior insular cortex, which in turn, promotes an increase in gray matter volume.

We also found a correlation between the parental praise score and two personality traits in children: conscientiousness and openness to experience. Our results are similar to those reported by Mesurado and Minzi (2013). They observed that children who believed that their parents were accepting had high levels of extraversion, agreeableness, conscientiousness, and openness to experience. Acceptance is one component of parental warmth or responsiveness [52]. The mechanism underlying the associations between parental praise and both conscientiousness and openness to experience is not clear; however, the behavioral results support the concept that parental praise has a positive effect on the development of personality traits in children.

This study has some limitations. First, we conducted a partial correlation analysis; therefore, we were only able to capture the correlation, not the causal relationship, between parental praise and brain structure in children. To elucidate a more detailed relationship, further research is needed, such as longitudinal studies that capture the amount of daily parental praise and developmental changes in children’s brains. Second, this study adopted only one question item to study parental praise. In other words, we might not have completely captured the features of parental praise. Accordingly, more multilateral assessments of parental praise are needed. Third, the parental praise question we used in this study was for parents and we did not assess how children perceived praise from their own parents. Therefore, further research should also focus on children’s feelings regarding their parents’ parenting styles.

In conclusion, this study provides evidence that parental praise is associated with the structure of posterior insular cortex in children. To the best of our knowledge, this is the first study to elucidate the relationship between a particular positive parenting behavior and brain structure in children. Our findings suggest that parental praise can influence the development of the brain structure in children, particularly in a region associated with emotional regulation and auditory processing.
